# Anisotropic Metamagnetic Spin Reorientation and Rotational Magnetocaloric Effect of Single Crystal NdAlGe

**DOI:** 10.3390/ma16072771

**Published:** 2023-03-30

**Authors:** Keunki Cho, Wonhyuk Shon, Jaehan Bae, Jaewoong Lee, Seungha Yoon, Jinhee Kim, Jong-Soo Rhyee, Beongki Cho

**Affiliations:** 1 School of Materials Science and Engineering, Gwangju Institute of Science and Technology, Gwangju 61005, Republic of Korea; 2 Green Energy and Nano Technology R&D Group, Korea Institute of Industrial Technology, Gwangju 61012, Republic of Korea; 3 Korea Atomic Energy Research Institute, Daejeon 34057, Republic of Korea; 4 Department of Applied Physics, Institute of Natural Sciences, Kyung Hee University, Yongin 17104, Republic of Korea

**Keywords:** rotational magnetocaloric effect, magnetocaloric effect, metamagnetic transition, NdAlGe

## Abstract

Magnetic anisotropy strongly influences the performance of the magnetocaloric effect. We investigated the magnetocaloric properties of the NdAlGe single crystal with I4_1_md structure. The temperature-dependent magnetization revealed significant anisotropic properties; stable antiferromagnetic transition at *T*_N_ = 6 K for *H*//a and meta-magnetic spin reorientation at low temperature (*T* ≤ 5 K) within an intermediate field (*H* = 2 T) for *H*//c. During the metamagnetic spin reorientation, the abrupt change of the magnetic entropy leads to a significant magnetocaloric effect with negative magnetic entropy change (∆*S*_M_) by −13.80 J kg^−1^ K^−1^ at *T*_C_ = 5.5 K for *H* = 5 T along the H//c axis. In addition, the antiferromagnetic state for H//a shows the inverse magnetocaloric effect(I-MCE) by positive entropy change ∆*S*_M_ = 2.64 J kg^−1^ K^−1^ at *T*_N_ = 6 K for *H* = 5 T. This giant MCE accompanied by the metamagnetic transition resulted in a significantly large relative cooling power (158 J/kg at *H* = 5 T) for *H*//c. The giant MCE and I-MCE can be applied to the rotational magnetocaloric effect (R-MCE) depending on the crystal orientations. NdAlGe exhibits rotational entropy change ∆*S*_c−a_ = −12.85 J kg^−1^ K at *T*_peak_ = 7.5 K, *H* = 5 T. With comparison to conventional MCE materials, NdAlGe is suggested as promising candidate of R-MCE, which is a novel type of magnetic refrigeration system.

## 1. Introduction

Magnetocaloric effect (MCE) refers to the access of thermal energy generated by the induction or removal of a magnetic field to magnetic materials. MCE is considered a promising replacement for the existing air refrigeration system because it is a solid-state refrigeration technology without using environmentally hazardous gases [1,2]. The primary objective of MCE research was to broaden the application temperature range to room temperature. V.K.Pecharsky et al. [3,4] demonstrated a large entropy change −∆*S*_M_~12 J kg^−1^ K^−1^ at room temperature in Gd_5_Si_2_Ge_2_ compound.

Recently, cryogenic MCE also been devoted to increasing the performance, reaching 2 K. EuTiO_3_ exhibited a substantial MCE performance of −∆*S*_M_ = 40 J kg^−1^ K^−1^ [5]. Furthermore, the current research in hydrogen storage technology involves liquefied hydrogen with liquefaction at low-temperature range, around 20 K [6]. The high MCE performance near the hydrogen liquefaction temperature can be applied to low temperature cryogenic cooling technology.

Recently, a new design of magnetic refrigerators has been proposed as a solution to conventional magnetic refrigeration’s inefficiencies, which is called the rotational MCE(R-MCE) system. In order to achieve magnetic cooling, it is necessary to subject the magnetocaloric materials to an uninterrupted process of magnetization and demagnetization while they are being rotated within a particular magnetic field region. Previous design of magnetic refrigerator inevitably contained an unnecessary empty space during in and out from the magnetic field region.

The R-MCE involves a cooling or heat removal system through the adiabatic rotation of a material relative to the applied magnetic field [7]. This system eliminates the empty space in traditional MCE designs and improves the efficiency of device space utilization. A material with strong magnetic anisotropy can exhibit a large R-MCE. To elucidate a large R-MCE, theoretical investigations suggested that spin ice with Kasteleyn transition can have a large magnetic torque [8]. R-MCE has also been observed in molecular magnetism [9].

The *R*Al*X* (*R* = La~Nd, *X* = Si, Ge) material group has been known as Weyl semimetal material candidates [10]. The NdAlSi exhibited helical magnetism mediated by Weyl fermion [11]. The PrAlGe showed a significant anomalous Hall effect due to nonzero Berry curvature [12]. In addition, a significant magnetic entropy change has been reported in the *R*Al*X* system. Liu, et al. reported ∆*S*_M_ = −19 J kg^−1^ K^−1^ in PrAlGe [13]. The critical exponents from ∆*S*_M_ and magnetic properties of the PrAlGe argue that it is a nearly theoretical second-order magnetic transition with 2D Ising spin model.

In this study, we present the magnetocaloric properties of NdAlGe, as a new materials platform for R-MCE. The helical spin structure of the NdAlGe shows a sizeable magnetic anisotropy due to the significant crystal anisotropy with I4_1_md structure. We investigate the MCE performance of high-quality NdAlGe single crystals. We confirm that the NdAlGe exhibits strong R-MCE properties, which is a promising candidate for practical R-MCE applications.

The significance of the R-MCE lies in its potential applications and advantages compared to the conventional MCE. First, devices utilizing the R-MCE can be more compact compared to those based on the MCE, as the magnetic field can be generated by rotating permanent magnets, which can be miniaturized and require less space than the electromagnets typically used in MCE-based devices. Second, The R-MCE can potentially reduce the power consumption of cooling devices, as rotating permanent magnets can generate a magnetic field without the need for continuous electrical input. This can lead to cost savings and more sustainable cooling solutions. Therefore, the finding of R-MCE properties of NdAlGe has the significance on the potential applications of revolutionize cooling technologies.

## 2. Materials and Methods

The single crystal of NdAlGe was synthesized using the Al self-flux method [10,11,12,13]. High purity elements of Nd (China Rare Metal Materials Co., Ltd., Chinese mainland, China, 99.9%), Al (Hydro, Oslo, Norway, 99.99%), Ge (Changsha Santech Materials Co., Ltd., Changsha, China, 99.99%) were placed in an Alumina crucible with a molar ratio Nd:Al:Ge = 1:10:1. The crucible was loaded into a quartz ampoule and sealed under high vacuum with 300 torr. The sealed quartz ampoule was heated to 1273 K for 12 h and slowly cooled from 1273 K to 1173 K in 5 K/h with the following 1.25 K/h cooling speed from 1173 K to 973 K. The samples were separated by spinning of centrifuge at 973 K. The one-step slow cooling from 1273 K to 973 K contains a NdAl_3_ impurity phase with a large needle shape. The two-step slow cooling is necessary to avoid the NdAl_3_ impurity.

Residual Al flux from the samples was removed by dissolving in a NaOH:H_2_O solution (volume ratio 1:5). The typical sizes of single crystals with shiny silver and flat surfaces were 3 × 3 × 1 mm. X-ray diffraction(XRD, Rigaku D/MAX-2500, Rigaku, Tokyo, Japan) measurements were performed using CuK*α* radiation (1.5405 Å) at room temperature. The stoichiometric ratio was verified by Energy Dispersive X-ray Spectroscopy with Field Emission Scanning Electron Microscope (FE-ESEM, Quanta 200 FEG, FEI, Hillsboro, OR, USA). Magnetic properties were measured by a Vibrating Sample Magnetometer option in Physical Properties Measurement System (PPMS Dynacool 14 T, Quantum Design Inc., San Diego, CA, USA).

## 3. Results and Discussion

### 3.1. Crystal Structure of NdAlGe

**Figure 1:**
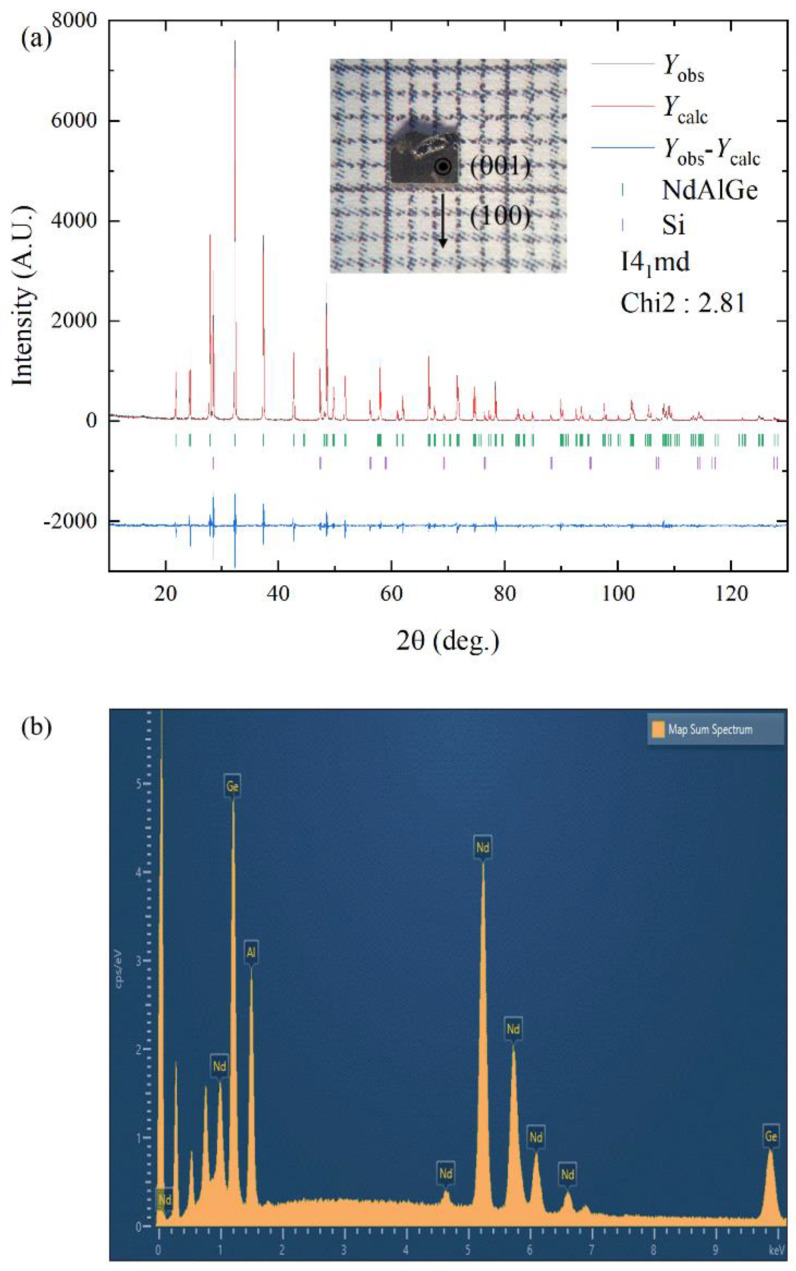
(**a**) Powder X-ray diffraction(XRD) of pulverized NdAlGe single crystals, inset: Crystal image and directions. (**b**) Energy dispersive X-ray spectroscopy (EDX) of NdAlGe.

The crystal structure of *R*AlGe (*R* = La~Nd) is I4_1_md (space group no. 109), which is a LaAlSi-type noncentrosymmetric structure [13]. Figure 1a shows the X-ray diffraction (XRD) pattern of NdAlGe, which is well matched with the I4_1_md structure using the Le Bail method. The wide flat surface of the samples is oriented along the [001] c-axis and the side edge of the crystals is the a-axis [100] from the XRD of the crystal surfaces.

The crystal structure of the NdAlGe has two plausible crystal structures; I4_1_/amd and I4_1_md, depending upon the Al-Ge anti-site defects [14]. From the crystal structure refinement, as presented in Table 1, the crystal structure of NdAlGe is closer to the I4_1_md rather than the I4_1_/amd because of a smaller standard deviation of I4_1_md (*χ*^2^ = 2.81) than those of I4_1_/amd (*χ*^2^ = 3.09). In the case of the I4_1_md crystal structure, it lacks a *m*_z_ mirror plane, thereby leading to an inversion symmetry breaking. We conducted energy dispersive X-ray spectroscopy (EDS) measurement of the NdAlGe, as shown in Figure 1b. The average elemental concentrations of Nd:Al:Ge by measuring six different points at the sample surface corresponds to 1:1.08:0.98, which is close to the pristine NdAlGe.

### 3.2. Magnetic Property of NdAlGe

**Figure 2:**
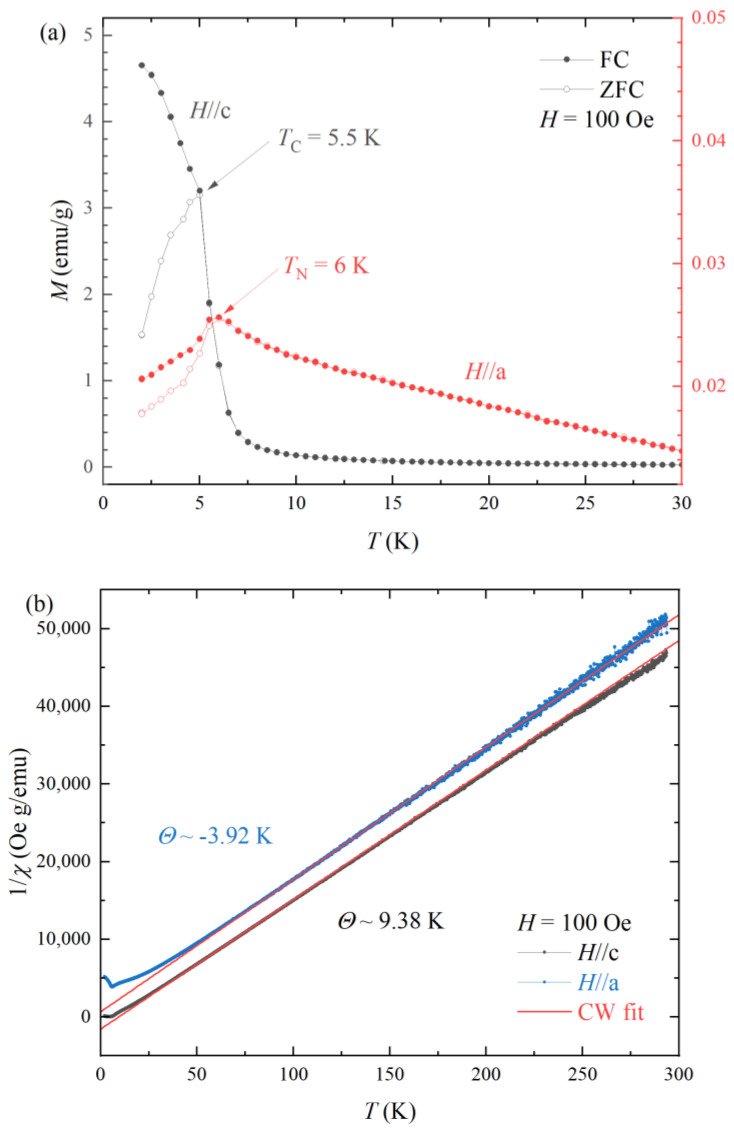
Temperature−dependent magnetization *M(T)* (**a**) and an inverse magnetic susceptibility *1/χ(T)* (**b**) with Curie−Weiss fit of NdAlGe.

Figure 2a exhibits the temperature-dependent magnetization *M*(*T*) with zero-field-cooling (ZFC, open symbol) and field-cooling (FC, closed symbol) thermal hysteresis under a static magnetic field of *H* = 100 Oe. The spin glass-like thermal hysteresis was found in other *R*AlGe compounds [15,16]. The divergence of FC and ZFC may be associated with the helical spin structure along the c-axis [12]. There are antiferromagnetic transitions at *T*_N_ = 5.5 K and 6 K for applying magnetic fields along the *H*//c and *H*//a, respectively. There is a sizable ZFC and FC divergence in thermal hysteresis for *H*//c. The magnetization for *H*//c (left axis) has two orders of magnitude larger than those of *H*//a (right axis), indicating the significant anisotropic magnetic structure. The *M*(*T*) of field-cooling for *H*//c seems to be a ferromagnetic ordering rather than an antiferromagnetic transition.

Figure 2b is the temperature-dependent inverse magnetic susceptibility *χ*^−1^(T) of FC data with Curie-Weiss fitting (line) at high temperatures. The Weiss temperature along the *H*//c corresponds to the positive value θ = 9.38 K, while the one of H//a shows negative antiferromagnetic Weiss temperature θ = −3.92 K, implying that the ferromagnetic interaction for *H*//c and antiferromagnetic interaction for *H*//a. The effective magnetic moment is estimated by *μ*_eff_ = 2.94 *μ*_B_ in both directions of *H*//c and *H*//a, which is in between those values of ionized Nd^2+^ = 2.68 *μ*_B_ and Nd^3+^ = 3.62 *μ*_B_, implying a possible mixed valence state of Nd^3+^ and Nd^2+^. The possible mixed valence state can be investigated as further research.

**Figure 3:**
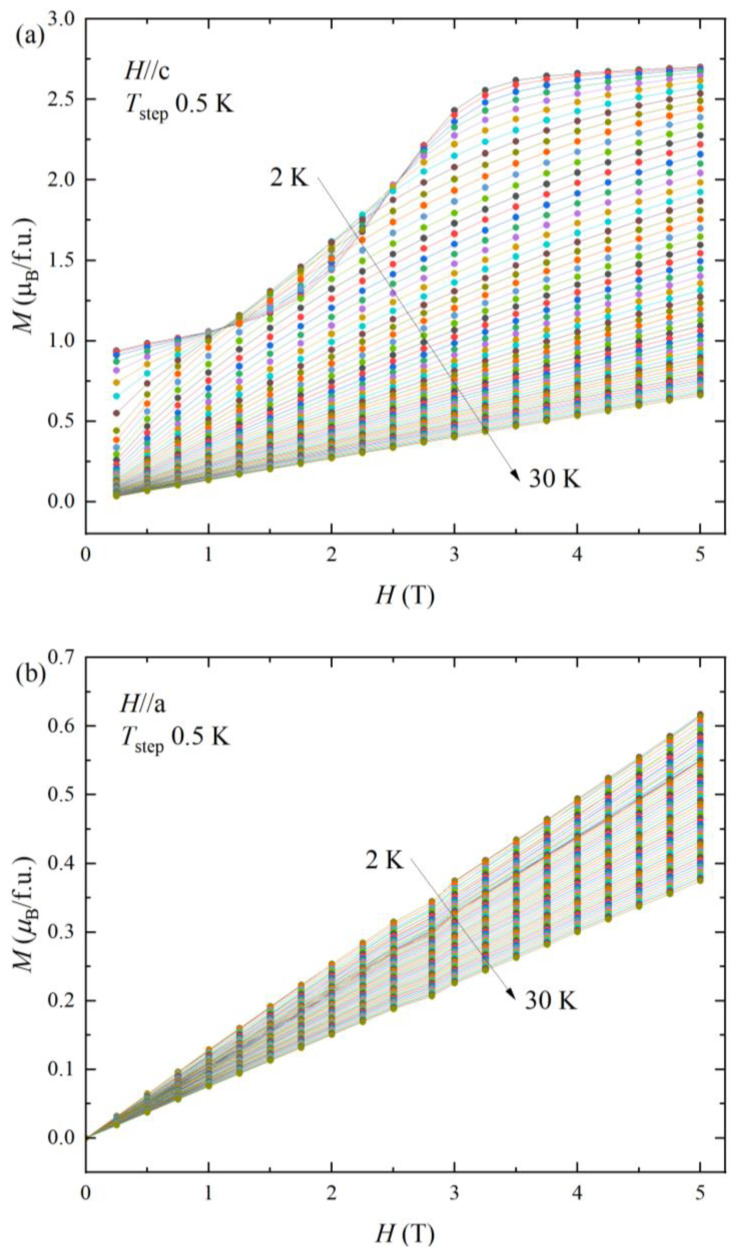
Isothermal magnetization versus applying magnetic field *M(H)* of NdAlGe along the *H*//c (**a**) and H//a (**b**).

The ferromagnetic nature is also observed in the isothermal magnetization with magnetic field *M*(*H*), as presented in Figure 3a. For the zero-magnetic field limit, the remanent magnetization is 0.9 *μ*_B_ at *T* = 2 K for *H*//c. Notably, there is a broad increase of magnetization over a broad range of *H* = 1~3 T and saturates to 2.75 *μ*_B_. The saturation magnetization is also in between the values of Nd^2+^ 2.4 *μ*_B_ and Nd^3+^ 3.27 *μ*_B_. The broad increase of magnetization is considered by a slowly varying spin reorientation. The remanent magnetization 0.9 *μ*_B_ at a low magnetic field is 1/3 of the saturation magnetization 2.75 *μ*_B_.

The NdAlSi compound showed a sharp metamagnetic transition near *H*_MT_ = 6 T along the *H*//c [12]. The neutron diffraction study demonstrates the incommensurate antiferromagnetic state (↑↓↓) at low field (*H* < *H_MT_*) to commensurate ferromagnetic state (↑↑↑) at high field (*H* > *H_MT_*), respectively [12]. Comparing with the sharp metamagnetic transition in NdAlSi, the NdAlGe shows a slow spin reorientation in a broad magnetic field range. The broad spin reorientation is not observed above *T*_C_ = 5.5 K. At high temperature T > 30 K, the magnetization curve follows a typical paramagnetic linear behavior. The magnetization versus magnetic field along *H*//a presents a typical antiferromagnetic increase with low magnetization values, as shown in Figure 3b, which is consistent with the stable antiferromagnetic transition for *H*//a of Figure 2a.

### 3.3. Magnetocaloric Effect of NdAlGe

The spin reorientation in the magnetization curve can make spin entropy change [17]. The significant anisotropic spin structures with ferromagnetic spin alignment for *H*//c and antiferromagnetic ordering for *H*//a are critical for a high magnetocaloric effect. The magnetic entropy change ∆*S*_M_ is calculated as the following relation:

(1) $$∆S_{M}=\int_{0}^{H}{\left(\frac{\delta M}{\delta T}\right)}_{H}dH$$

**Figure 4:**
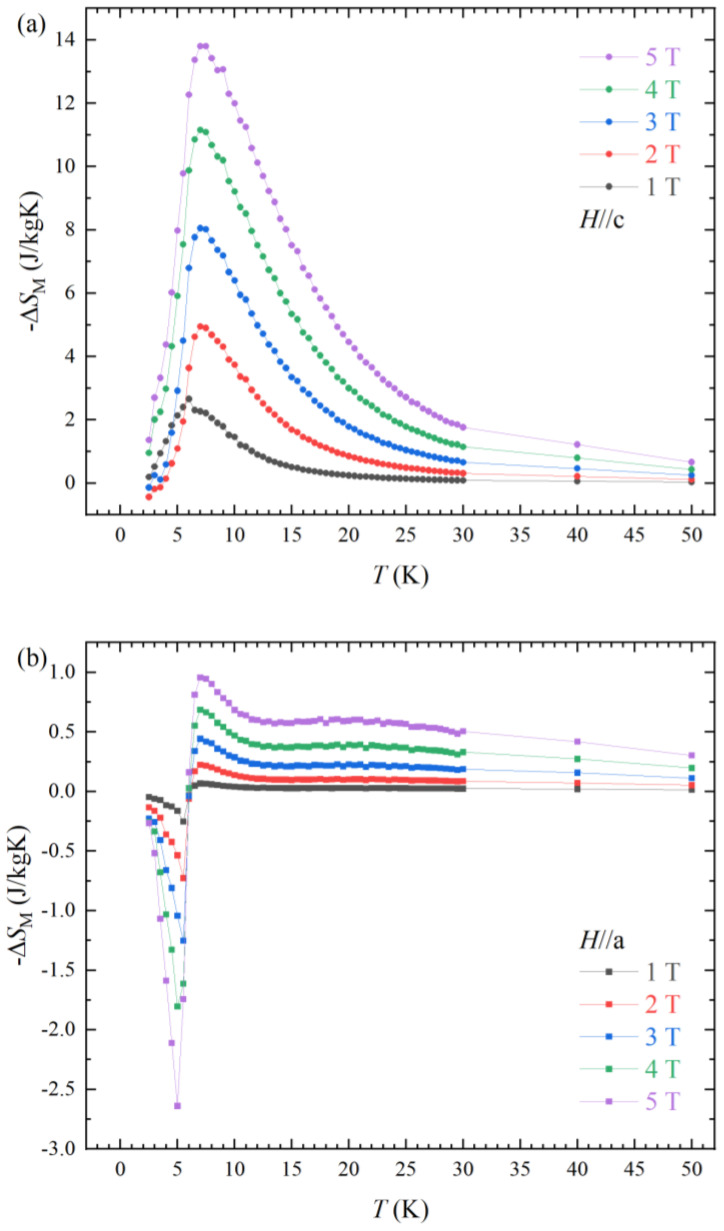
Entropy change −∆*S*_M_ of NdAlGe along the *H*//c (**a**) and H//a (**b**) under various magnetic fields, as indicated.

The calculated values of the ∆*S*_M_ are depicted in Figure 4. The ∆*S*_M_ for *H*//c in Figure 4a reveals a considerable MCE value −∆*S*_M_ = 13.8 J kg^−1^ K^−1^ at *H* = 5 T and *T*_peak_ = 7 K (*T*_peak_; temperature of ∆*S*_max_). On the other hand, the entropy change in the antiferromagnetic state for *H*//a presents a positive ∆*S*_M_ value ∆*S*_M_ = 2.64 J kg^−1^ K^−1^ at *H* = 5 T, *T*_peak_ = 6 K, indicating the inverse magnetocaloric effect (I-MCE). The maximum peak temperature *T*_peak_ of −∆*S*_M_ is a little bit increased from 6 K (*H* = 1 T) to 7 K (*H* = 2 T), which is attributed to the metamagnetic spin reorientation. The *T*_peak_ is not changed for higher magnetic field ranges (*H* ≥ 2 T). The full width-half maximum of −∆*S*_M_ also increased for higher magnetic fields, indicating that the metamagnetic spin reorientation under high magnetic field enhances the −∆*S*_M_, *T*_peak_ and cooling power. *T*_peak_s of MCE and I-MCE are higher than the values of critical temperatures *T*_C_ = 5.5 K and *T*_N_ = 6 K for *H*//c and *H*//a, respectively. The different peak temperatures and the temperatures of magnetic ordering can be accounted by the 3D Heisenberg model and tricritical mean field model [18,19].

### 3.4. Rotational Magnetocaloric Effect of NdAlGe

**Figure 5:**
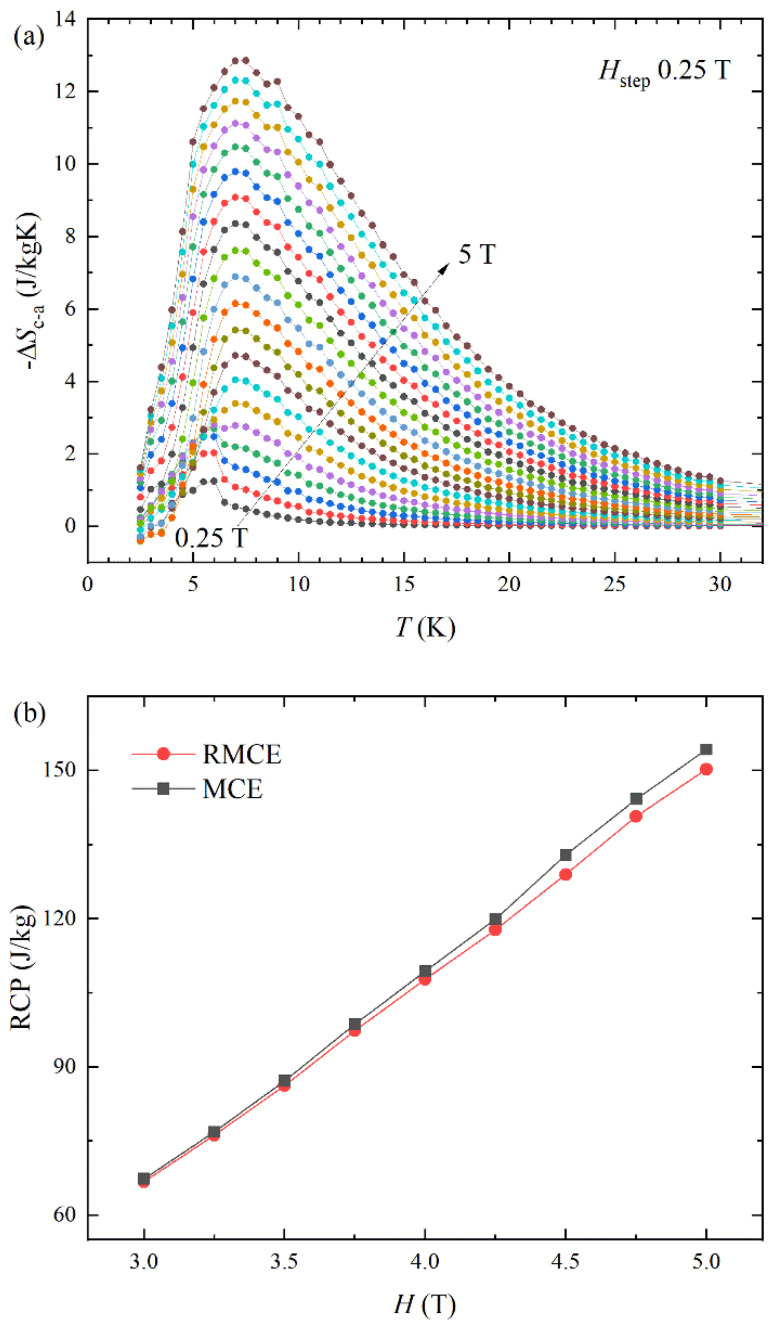
Rotational magnetocaloric effect (R-MCE) −∆*S*_c−a_ (**a**) and relative cooping power (RCP) of MCE and R-MCE (**b**) of NdAlGe.

The anisotropic magnetic structure with ferromagnetic and antiferromagnetic states along the *H*//c and *H*//a-axis results in a significantly different spin entropy change. The anisotropic spin entropy change possesses the promising R-MCE property. Figure 5a presents the R-MCE property with a temperature of NdAlGe under various magnetic fields. The rotational spin entropy change is defined by ∆*S*_R_ = ∆*S*_c−a_ = ∆*S*_c_ − ∆*S*_a_ [20,21]. The NdAlGe exhibits relatively large R-MCE performance, as shown in Figure 5a, due to sign reversal between MCE and I-MCE for different crystal orientations. The ∆*S*_R_ exhibits a very large value ∆*S*_c−a_ = −12.85 J kg^−1^ K^−1^ at *T*_peak_ = 7.5 K *H* = 5 T. The ∆*S*_R_ enhances over a wide temperature range compared to the ∆*S*_M_ of *H*//c.

The enhancement of entropy change over a wide temperature range is critical for large relative cooling power (RCP). The RCP is calculated by RCP = ∆*S*_max_δ_FWHM_, where the δ_FWHM_ is the full-width-half-maximum (FWHM) of the ∆*S*_R_ or ∆*S*_M_. Figure 5b presents the RCP of MCE and R-MCE cases. It can be seen as the performance of R-MCE is comparable to that of conventional MCE for the c-axis.

In general, isotropic MCE materials have a negligible value of R-MCE due to the comparable ∆*S*_M_ in both different crystal orientations. The comparable RCP value of the R-MCE spin entropy change with those of MCE suggests the substantial implications of practical applications on R-MCE type refrigeration system.

### 3.5. Arrott Plot and Magnetic Transition Order Comparison

From the thermodynamic Maxwell relations, the first-order magnetic transition (FOMT) is critical to high MCE performance and the second-order magnetic transition (SOMT) is beneficial to significant entropy change over a wide temperature range [22,23]. So, we analyze the magnetic transition analysis in terms of Arrott plot [20]. The critical exponents of magnetization *M* and magnetic field *H* with temperature have the following relation:

*H*^1/*γ*^ = *a*(*T* − *T*_C_)*M*^1/*γ*^ + *bM*^1/*β*+1/*γ*^ (2)

where *β* and *γ* are the critical exponents. In the vicinity of Curie temperature *T_C_*, Landau free energy expansion by assuming a small magnetization *M* becomes minimum when it satisfies the following relation.

(3) $$\mu_{0}H=aM+bM^{3}$$

where *β* =1/2 and *γ* = 1 in the critical exponents relations. From the previous report [20], the scaling exponents of the NdAlGe are obtained by *β* = 0.236 and *γ* = 0.92, which is close to the tricritical mean field theory. Therefore, it is reasonable to use the critical exponents with the simplified model of tricritical mean field values.

**Figure 6:**
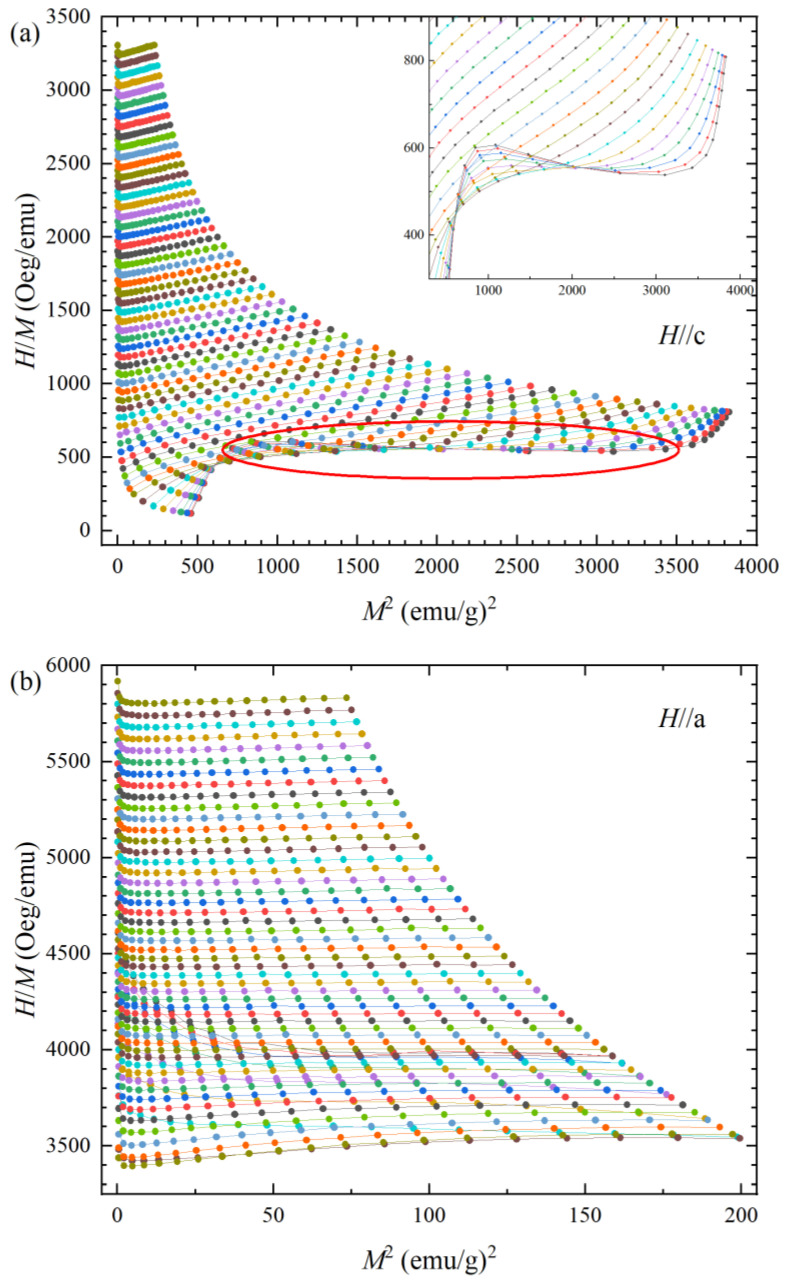
Arrott plots *H*/*M* versus *M*^2^ of NdAlGe *H*//c (**a**) and *H*//a (**b**).

Figure 6a represents the Arrott plot *H*/*M* versus *M*^2^ for *H*//c. As the induced magnetic field increases, a positive slope is observed over a wide field regime. The positive slope becomes negative during a slow spin reorientation region, as marked with a red circle in Figure 6a. The positive slope in the Arrott plot indicates the second-order magnetic transition (SOMT). The negative slope region indicates that the spin reorientation is the first-order magnetic transition (FOMT). The FOMT exhibits a significantly higher maximum value of ∆*S*_M_, albeit with a relatively narrow full width and half maximum *δ*_FWHM_. Consequently, materials exhibiting FOMT may possess an average or lower RCP [21,24]. In contrast, SOMT materials exhibit a relatively broad *δ*_FWHM_ and a lower maximum ∆*S*_M_ than those of FOMT. Typically, the SOMT materials have small magnetic hysteresis, offering the advantage of better MCE performance.

The consistent positive slope in the Arrott plot indicates that the temperature-dependent magnetization of the material is in the second-order magnetic transition (SOMT). However, the presence of a metamagnetic spin reorientation induces a shift to a negative slope, indicating a first-order magnetic transition (FOMT). The alteration in transition order implies a fundamental difference in the MCE performance. The FOMT usually exhibits a significantly higher maximum value of ∆*S*_M_. The first elucidation of MCE by A.O. Pecharsky [4] also observed FOMT of the Gd_5_Si_2_Ge_2_, leading to the highest MCE performance at room temperature. In contrast, SOMT materials exhibit a relatively broad *δ*_FWHM_ but lower maximum of ∆*S*_M_. Therefore, the coexistence of FOMT and SOMT has a synergy to increase the entropy change as well as broaden the operating temperature range due to the large temperature width *δ*_FWHM_.

The first-order magnetic transition accompanies magnetic hysteresis behavior, which renders fatigue during the magnetization process. On the other hand, the FOMT of the NdAlGe does not show magnetic hysteresis, indicating that there is no fatigue on the process. In addition, the NdAlGe compound demonstrates a broad *δ*_FWHM_ due to the SOMT on the overall induced magnetic field range. The FOMT without magnetic hysteresis on the compound is attributes to the increase of the entropy change, while the SOMT widens the operating temperature range and RCP value. Therefore, the anisotropic magnetic properties and distinctive phase transition behavior of the compound have a significant contribution on the practical applications of R-MCE.

### 3.6. Performance of NdAlGe Compared with Other Materials

**Figure 7:**
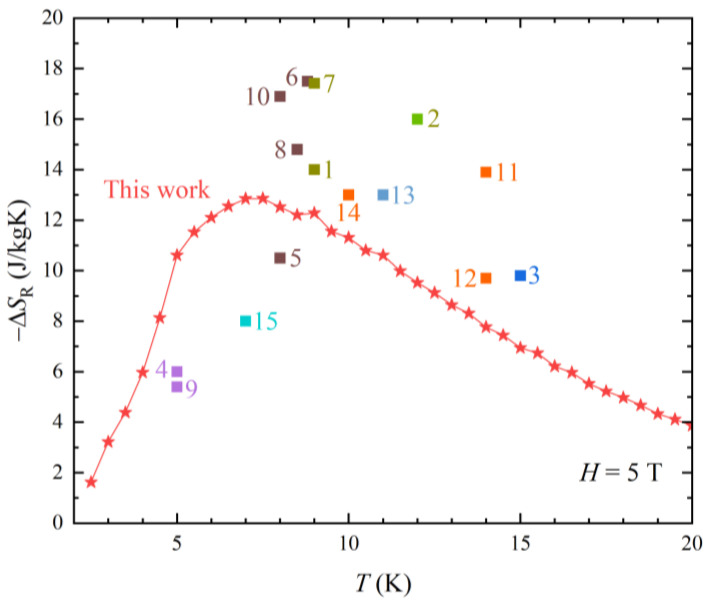
Temperature dependent R-MCE −∆*S*_R_ of NdAlGe and other single materials, ever reported.

The performance of R-MCE in NdAlGe is evaluated by comparing other materials. As presented in Figure 7 and in Table 2, the NdAlGe exhibits a *T*_peak_ = 7.5 K and −∆*S*_R_ = 12.86 J/kgK, which positions it compatibly with the *R*CrO_3_ group with equivalent magnetic field strength (*H* = 5 T). Because the *R*AlGe compounds are regarded as a represented large R-MCE group, the NdAlGe can be a candidate for the high R-MCE materials. When we investigate *R*Al*X* (*R* = rare earth, *X* = Si, Ge) materials system with strong anisotropy, we can find a material with superior R-MCE performance, which can be applied to a novel approach to MCE-based applications, such as H_2_ liquefaction.

## 4. Conclusions

A single crystal of NdAlGe with a noncentrosymmetric I4_1_md structure was synthesized by self-flux method. Magnetic measurements revealed that NdAlGe exhibits a ferromagnetic (FM) structure for *H*//c in field cooling and an antiferromagnetic (AFM) structure for *H*//a. In the case of *H*//c, we observed a metamagnetic spin reorientation near *H* = 2~4 T below 5 K. The Arrott plot results confirmed that the metamagnetic spin reorientation in NdAlGe is the FOMT (negative slope in Arrott plot), while there are SOMT type positive Arrott plot in other temperature and field ranges. Because the FOMT increases entropy change and the SOMT expands the operating temperature range, the coexistence of the transition order type is beneficial to the enhancement of MCE performance over a wide temperature range.

Based on the results of ∆*S*_M_ calculation, we observed a large MCE of NdAlGe corresponding to −3.8 J kg^−1^ K^−1^ at *T*_peak_ = 7 K in the c-axis. The sign in the a-axis was flipped to positive I-MCE, resulting in a sizable R-MCE performance at *T*_peak_ compared to previously reported compounds. Consequently, NdAlGe shows potential as an R-MCE material at cryogenic temperatures, which is required for various technologies such as hydrogen liquefaction.

## Figures and Tables

**Table 1:** Crystal structure of NdAlGe.

	NdAlGe	Nd(Al_2_Ge_2_)_0.5_
a (Å)	*I*4_1_*md*	*I*4_1_*/amd*
b (Å)	4.2358	4.2374
c (Å)	14.6445	14.6499
*χ* ^2^	2.81	3.09

**Table 2:** R-MCE of single materials in Figure 7.

R-MCE Single Materials	*T*_peak_ (K)	−∆*S*_R_ (J/kgK)	Ref.
NdAlGe	7.5	12.86	This work
1. DyMnO_3_	9	14	[25]
2. TbMnO_3_	12	16	[26]
3. HoMn_2_O_5_	15	9.8	[27]
4. ErGa_2_	5	6	[28]
5. HoGa_2_	8	10.5	[28]
6. DyNiSi	8.8	17.5	[28]
7. TbFeO_3_	9	17.42	[29]
8. DyCrO_3_	8.5	14.8	[30]
9. TbCrO_3_	5	5.4	[30]
10. ErCrO_3_	8	16.9	[30]
11. HoNiGe_3_	14	13.9	[31]
12. ErAlO_3_	14	9.7	[32]
13. KEr(MoO_4_)_2_	11	13	[33]
14. Tb_2_CoMnO_6_	10	13	[34]
15. TmB_4_	7	8	[35]

## Data Availability

Data available on request due to restrictions eg privacy or ethical.
